# Assigning Punishment: Reader Responses to Crime News

**DOI:** 10.3389/fpsyg.2022.784428

**Published:** 2022-02-16

**Authors:** Kat Albrecht, Janice Nadler

**Affiliations:** ^1^Department of Criminal Justice and Criminology, Andrew Young School of Policy Studies, Georgia State University, Atlanta, GA, United States; ^2^Northwestern Pritzker School of Law, American Bar Foundation, Chicago, IL, United States

**Keywords:** blameworthiness, homicide, punishment, crime news, political ideology

## Abstract

In this study we test how the composition of crime news articles contributes to reader perceptions of the moral blameworthiness of vehicular homicide offenders. After employing a rigorous process to develop realistic experimental vignettes about vehicular homicide in Minnesota, we deploy a survey to test differential assignments of suggested punishment. We find that readers respond to having very little information by choosing neutral or mid-point levels of punishment, but increase recommended punishment based on information about morally charged conduct. By contrast, information about the perpetrator’s immigration status caused respondents to split into two groups on whether the offense deserves neutral or increased punishment. We find that political ideology strongly influences recommendations for more severe punishment when the immigration status of the perpetrator is revealed. We argue that this difference represents a moral dimension to punishment and blameworthiness that incorporates factors outside the active offense and therefore reveals the social influence of differential reporting in shaping public perception.

## Introduction

The content and construction of crime news provides an important resource for examining social inequality. American media produces a large quantity of news about crime, and this reporting resonates with Americans (Boulahanis and Heltsley, 2004; Norman, 2018). Importantly, the news is not a monolith; instead, it is a shared universe of interactive creation, allowing us to digest information from the world around us and extract value from it (Pan and Kosicki, 1993; Berkowitz, 1997; Lu, 2012). News shapes our perception of the world – not by providing an objective reflection of facts, but rather by filtering information through a lens of news creation constructed by news reporters (Schudson, 2011). By studying the filtering process through which information becomes news stories, we can understand how readers form beliefs and opinions about guilt and innocence in crime news.

In this study we analyzed how the construction of news stories can change the perceptions of news readers. Specifically, we tested how altering both the quantity and the nature of the information presented can change perceptions of blameworthiness and punishment. First, we conducted a detailed content analysis of homicide news articles in Minnesota to develop three news vignettes that cue different levels of moral culpability of vehicular homicide offenders. Next, we conducted a survey experiment using the news vignettes to measure perceptions of punishment. We observed differing punishment recommendations that varied according to political views and other demographic factors. The results suggest a link between news and the current political climate, specifically invoking beliefs about morality as guiding belief in punishment.

## The Importance of Crime News

Newspapers do not exist in a vacuum; they are created for and digested by an audience who themselves exist in the social world. Thus, the flow of information from news media is not uni-directional; rather it is a socio-cognitive relationship involving multiple actors. Pan and Kosicki (1993) describe the shared cultural universes of sources, journalists and audiences in the dissemination of news media with particular emphasis on the role of the audience as both readership and financial life-force for the institution of news. Shoemaker (2006) explains the logistics of this system of news and the interactive roles of its constituents.

“News is a commodity. It can be bought, sold, and traded. Journalists manufacture the news. Public relations firms manipulate the news. The audience consumes the news. Advertisers pay to place their products next to the news. News travels by word of mouth, across the Internet and other mass media. Professional associations focus on the production of news and on social science research about news. Televised news shouts at us in airport waiting rooms. News is ubiquitous” (106).

These tensions are not about fabricating news, but rather characterize news as a social institution shaped by economics, technology, politics, culture, and organizational structures (Schudson, 2011). This perspective helps us transcend the logistical process of reporting news and instead intuit value from its actual construction (see Berkowitz, 1997; Lu, 2012). Fishman (1988) argues that the news is in fact socially constructed, employing the example of a 1976 crime wave against elderly New Yorkers. This particular crime wave, while made up of real criminal incidents – was not actually an increase in crime from the same period in the previous year. Fishman explained that reporters did not fabricate the news, rather “they gave a determinate form and content to the incidents they report(ed)” (1988:10–11). This explanation gives reporters greater status than inscribers of rote fact – instead they interpret and ascribe meaning to events in the way that they report them. Indeed, reporters are quite cognizant of the social meaning of the events they report about even though news is very subjective (Gieber, 1964). The shaping of news is important because of its influence in the everyday lives of consumers. Ninety-three percentage of Americans say they follow the news at least occasionally, a large majority of them reporting that they do so for reasons that are primarily due to social interactions and civic responsibility (Purcell et al., 2010). In this way, the very circulation of news is dependent on the same society it reports about.

Crime news is one of the most prevalent types of reported news, but numerous studies have concluded crime news does not correlate with actual crime rates (Graber, 1979; Dorfman et al., 2001; Boulahanis and Heltsley, 2004). For example, a 2001 study of crime reports in the LA Times concluded that 80% of murders were reported on, but only 2% of physical and sexual assaults received news coverage (Dorfman et al., 2001). This creates a news-scape where some crime news is disproportionately reported, with a particular emphasis on murders. While the sheer volume of crime reporting as a percentage of space may be high, researchers conclude that this deluge of crime content may actually be keeping pace with the readerships desire to read about crime (Graber, 1979). So, in this sense, news about crime is reported to the same extent that readers want to read about crime rather than in proportion to its actual occurrence.

The prevalence and construction of crime news matters because of its connection to negative consequences on attitudes, including racial stereotyping, public mis-perceptions of certain people as super-predators, and fostering fear of crime that does not accurately reflect the real spatial/demographic picture of crime (Barlow et al., 1995; Gilliam et al., 1996; Sorenson et al., 1998; Thorson, 2001; Boulahanis and Heltsley, 2004). These effects are attributable not only to the simple dichotomy of which cases are covered and which ones are not, but also to the way in which cases are covered and constructed. In one study, researchers found that the way news is reported implies that minority persons, unemployed persons, and male youths are more often members of deviant social groups (Humphries, 1981; Meyers, 2004; Dixon, 2006).

One theory about variation in reporting focuses on the concept of newsworthiness and efforts to make content newsworthy. Surette (1998) usefully defined newsworthiness as essentially “…the criteria by which news producers choose which of all known events are to be presented to the public as news events (60).” Chermak (1995) presented some of the earliest evidence that news reporters consciously select crime stories for reporting based on how newsworthy they were. Importantly, Chermak noted that not only are not all crimes newsworthy, even some extreme crimes like homicide were deemed “not interesting enough” to be covered by the media (1998). This further illustrates the shared space of journalist and reader where anticipated reader response can help drive reporting decisions.

Katz (1987) proposes that for something to be newsworthy it must transgress a moral boundary as internalized by society. Increased attention to crime news can produce harsher blameworthiness evaluations for Black suspects compared to White suspects (Dixon, 2008), demonstrating that boundaries of morality are subject to and derivative of other biases in society. This poses difficult and important questions for why certain victims are more sympathetic and certain offenders are perceived as guiltier. We explore these questions here through the lens of criminal law, using vignettes designed to trigger moral judgments, such as drunk driving and illegal immigration.

## Blameworthiness and Criminal Law

Psychological judgments about blame rely on both the harm that the agent causes as well as the mental state of the agent at the time she caused the harm (Cushman, 2008). Thus, two friends who walk out of a bar and who each crash while driving home in the snow are blamed differently depending on the harm they cause. We blame and punish more severely a drunk driver who injures a person than a drunk driver who damages a tree, even if all else is equal (Cushman, 2008). In addition, we blame and punish a person who intentionally causes harm more severely than a person who unintentionally causes the exact same harm (Alicke and Davis, 1989; Alicke et al., 1994; Robbennolt, 2000). These psychological judgments arise from intuitions about blame and punishment, including attitudes about how severely to punish and for what purpose (Carlsmith et al., 2002; Carlsmith and Darley, 2008; Pizarro and Tannenbaum, 2012; Bilz, 2016).

At the same time, much blame and punishment occur within a social context, imposed by institutions and subject to guidelines or regulations. Governments, schools, firms, and the like typically have formal blame and punishment systems – formal rules are enforced by designated individuals, and the process is highly institutionalized (Cushman, 2014). At the same time, institutionalized blame and punishment relies heavily on our intuitive sense of justice (Robinson and Darley, 1995; Mikhail, 2007). The prototypical example of regularized blame and punishment is the criminal legal system. In criminal law, blameworthiness is codified into law by a set of standards that include the consideration of mens rea, or guilty mind, and actus reus, or wrongful act. Historically derived from Christianity, generally immoral conduct was sufficient to prove mens rea (Robinson, 2002). By the middle of the 13th century, it was well established that “justifiable punishment is premised on and proportional to moral guilt” (Gardner, 1993; p. 655). Historically, punishment was thus intrinsically connected to moral blameworthiness, and contemporary philosophical conceptions of punishment include moral responsibility as a central condition for punishment (Bennett and Brownlee, 2020). While current systems of criminal law have developed into a less explicitly normative inquiry into the offender’s state of mind (Nadler, 2022), even contemporary conceptions of mens rea reflects the attachment of moral blame and the offender’s state of mind at the time of the offense (Gardner, 1993; Nadler, 2022). Blameworthiness intuitions continue to influence our justice system not only in assigning guilt, but also in prescribing punishment. The degree of resulting harm influences judgments of punishment as well as the perceived wrongfulness of the act, although the magnitude of the resulting influence is the subject of some debate (Cushman, 2008; Kneer and Machery, 2019). Severity of harm does not solely determine punishment, of course – for example, some homicides are punished less severely than others – even if the outcome of death is the same. We see this frequently in the contemporary justice system where we distinguish justifiable and non-justifiable killings, but also divide non-justifiable killings into degrees that call for less punishment based on less intent and mitigating circumstances.

Assessments of severity of harm, the actor’s role in causing or contributing to the harm, and the actor’s intentionality are not made in a vacuum. Often, judgments of these aspects of an actor’s role are made under uncertainty: How much intent did the actor have? How strongly causal was the actor’s role in the harm? Alicke’s (2000) theory of culpable control posits that when people assess blame, they try to assess how much control the actor exercised over the harm. If an actor intentional conduct directly causes the harm, then the actor is perceived to have high control. But under uncertainty, these perceptions of intent and harm are directly influenced by our initial affective reaction to the harm situation. For example, if John crashes while speeding home to hide an anniversary present for his parents, he is judged less harshly than if he is hiding a vial of cocaine he left out in the open, even though the harm (injuring another driver) and the intentionality (less than intentional, but unreasonably disregarding risk) is the same in both scenarios (Alicke, 1992; see also, Nadler, 2012; Nadler and McDonnell, 2012). John-the-cocaine-hider evoked stronger initial affective reactions, which motivated a desire to understand the conduct as more blameworthy than that of John-the-present-hider. On this account, we engage in “blame validation” – we make blame attributions spontaneously according to how strongly negative our gut reaction is, and then we validate our blame assessment by adjusting evaluations of intention and causation accordingly.

The standard theoretical inputs for punishment and blame judgments – such as intent and severity of harm – are therefore themselves influenced by our perceptions of what kind of person the actor is, including the actor’s motives for acting and her character (Uhlmann et al., 2015; Siegel et al., 2017). Alicke’s culpable control model posits that we constantly evaluate other people to determine which individuals are trustworthy in the sense of promoting rather than threatening our own physical and psychological well-being (Hieronymi, 2004; Alicke, 2014). According to person-based theories of moral blame, we spontaneously evaluate wrongdoing based on features of the person before having the opportunity to carefully weigh the legally central features of mental state and resulting harm. Evaluating features of the person might include legitimate considerations of motive (e.g., a person driving through a red light to rush someone to the hospital is legitimately blamed less for causing harm than a person engaging in the same conduct to show off for friends). But less legitimate features of the person also influence perceptions of blame, intentionality, and causal role in harm, such as perceived moral character (Nadler, 2012; Nadler and McDonnell, 2012). And other features of the person are completely illegitimate (such as race, national origin, religion) but might nevertheless influence blame and punishment judgments *via* the culpable control pathway posited by Alicke (2000, 2014).

Blame by nature relies on causal responsibility by a human agent, and so invokes a judgment of responsibility that is moral in nature (Coates and Tognazzini, 2012). For this reason, the conduct to which we attach blame reflects poorly on the actor as a moral agent and leads us to infer moral character that lacks loyalty, integrity, or the like (Coates and Tognazzini, 2012). At the same time, prior judgments of moral character can themselves influence degree of blame, as we just discussed.

In the studies reported here, we test the effect of two such person-based factors – one legitimate and one illegitimate – on perceptions of blame and punishment. We do this by cuing morality in vignettes about drinking and driving and illegal immigration, which we describe in further detail below the section “Site of the Research.” Moral Attitudes, Blame, and Punishment When an agent causes harm in a context that the public views as morally objectionable, people view the conduct causing harm in a negative light. We saw this earlier in the vignette about John-the-cocaine-hider. Because possession and use of illegal drugs is viewed by many as morally objectionable, John’s conduct that led to the accident was viewed negatively. At the same time, when the agent is a member of certain social outgroups (for example, homeless people, undocumented migrants), that agent is viewed as less competent and trustworthy and their conduct more blameworthy (Fiske, 2018). We next develop examples of morally objectionable conduct (drunk driving) and a morally derogated outgroup (immigrants) that we use to form the basis of the experimental study on assigning punishment that we report below.

### Drunk Driving and Moral Attitudes

Fifty years ago, the decision to get behind the wheel of a car after drinking alcohol was considered mostly a matter of personal preference. In the ensuing years, the issue of driving while impaired by alcohol underwent a radical change and moved into the domain of morality. During the 1980s, activists grew the number of local anti-drunk-driving groups from a few dozen to over 400. Their goal was to reduce drunk driving in their respective communities (McCarthy and Wolfson, 1996). Aided by national umbrella organizations, local activists focused on moralization of the issue with the message “You can make a difference” – a slogan plainly designed to appeal to the American ethic of individual responsibility. At the same time, the success of the effort to move drunk driving into the consciousness of the public and into the domain of the moral depended on tapping into and managing intense emotions, like fear. Mothers Against Drunk Driving (MADD) is the highest profile organization of its kind in the United States, and its very name evokes the tragic image of a mother grieving for a dead child, “a threat to something sacred in society: the relationship of mother and child…” (Schmidt, 2014).

The fear of a drunk driving crash in the future presents the looming potential of losing one’s own life, losing a loved one, or taking another person’s life (Schmidt, 2014). Drunk driving injuries and deaths are shaped into narratives involving a binary moral discourse involving immoral, anti-civil perpetrators acting upon innocent victims. Collectively the acts performed by these individual perpetrators – driving vehicles while under the influence of alcohol – represent a challenge to the moral foundations of society (Schmidt, 2014). At the same time, because drunk driving is a behavior that is ongoing and strikes randomly, there is the possibility that any one of us could become a victim in the future.

Perpetrators of drunk driving accidents are framed as individuals who make a choice: they put the key in the ignition. By choosing to insert the key, the individual is portrayed as choosing not to care about others and instead to put them at risk – a fundamental lack of compassion. The MADD narrative presses us to empathize with the anguish of a mother whose young adult child’s life has suddenly ended. The individual who chooses to insert the key after drinking is portrayed as displaying a complete disregard for that anguish. By disregarding this pain and sorrow, the drunk driver is perceived as rejecting this sacred value of motherhood and is rendered a moral monster.

Strong moral reactions can result from harm that is diagnostic of the actor’s moral character. For example, a CEO who spent company funds redecorating his office while the company was cutting thousands of jobs provoked public scorn not because the act of redecorating was particularly harmful but because in context the act was seen as indicative of the CEO’s character (Tannenbaum et al., 2011). When evaluating wrongs and harmful acts, people care about what kind of person the actor is: who that person is and not just what they have done (Nadler, 2012; Nadler and McDonnell, 2012). Certain acts are viewed as highly informative of character: these include animal cruelty, racist speech, and to some extent in recent decades, drunk driving, especially when it results in injury or death.

### Moral Attitudes Toward Immigrants

In the past few decades, immigration patterns in the United States shifted such that immigrants now live in communities throughout the nation, rather than being concentrated in a handful of regions. Many Americans have negative attitudes toward immigrants as a group – most commonly that immigrants cause problems and should be kept out of the country. At the same time many people hold positive attitudes toward immigrants, including the belief that they are hard-working and enrich American culture. Sometimes these conflicting negative and positive views are held by the same individuals (Ostfeld, 2017). White Americans’ attitudes toward immigrants tend to track with their racial attitudes, and individuals who hold more ethnocentric views are more hostile toward immigrants who come from countries outside of Europe (Hainmueller and Hopkins, 2014). Racially resentful whites would like to see restrictions on the flow of immigrants as well as government services denied to immigrants (Kinder et al., 1996; p. 123). Immigrants who entered the country without authorization are viewed negatively, especially by ideological conservatives (Hainmueller and Hopkins, 2014).

Racial resentment among whites increases when the presence of non-whites is perceived to affect their own community. “In the view of many Whites, Blacks in the neighborhood threaten property values and safe schools; Blacks at church violate definitions of community; Blacks at work stir up apprehensions about lost jobs and promotions. At the same time, distance from Blacks allows Whites the luxury of expressing racial tolerance” Kinder and Mendelberg (2000; p. 404). Experimental work has demonstrated that whites are less comfortable with immigrants living near them, working with them, and marrying into their family when those immigrants are depicted as darker skinned compared to when they are depicted as lighter skinned (Ostfeld, 2017). This finding was independent of whether the individual immigrants in question were more assimilated or less assimilated in American culture.

There is a significant literature discussing the morality of immigration, with a particular emphasis on illegal immigration. Importantly, scholars argue that illegal immigration is not always morally wrong depending on the larger belief structures and the incompatibility of multiple legal, social, and protective obligations. For example, if a country limits immigration more than it morally should, the illegal immigration may be a legitimate response rather than a moral breach (Risse, 2008; Taylor, 2008). Many of these writings in law and philosophy tie the moral obligation back to the state, but there is less work analyzing how a layperson in America might interpret the morality of illegal immigration. We do know that Americans are divided on the issue of illegal immigration and that ways of framing illegal immigration as an issue vary across the country. Discourse in border adjacent regions tends to focus on illegality in immigration (as opposed to immigration more broadly) and to be significantly racialized (Branton and Dunaway, 2009; Ramakrishnan et al., 2010; Merolla et al., 2013). Much of this framing plays out in the news, with different rhetoric and framing characterizing liberal/progressive versus conservative news sources (Merolla et al., 2013), though the changes in laypeople’s decision making as a result of those frames is less studied.

## Site of the Research

In this study, we survey readers in state of Minnesota in the United States due to a confluence of salient situational factors and a more general need for increased homicide research outside the largest urban settings^1^. First, we prioritized a location with a relatively high rate of occurrence of vehicular homicides, but that had varied sentencing outcomes. According to the Minnesota Sentencing Commission, while the sentencing guidelines under MN Statute 609.2112 recommend up to 10 years in prison for all vehicular homicide offenders, a substantial portion of vehicular homicide offenders receive stayed sentences or local confinement for a relatively short period (Minnesota Sentencing Guidelines Commission [MSGC], 2016/2017). This wide range primed readers with the realistic ability to make varied choices in punishment outcomes. Second, we chose a location with a standardized type of media coverage, i.e., one main news outlet that covers criminal news across the region. This increases the likelihood that participants will have seen news disseminated in a similar format.

## Data and Methodology

This study had two phases of data collection: the purpose of the first phase was to understand the standard formulation of news articles about Minnesota homicides, and in the second we constructed and deployed a vignette experiment. The survey experiment was designed to assess how readers assign punishment to perpetrators along two different dimensions – characteristics of the person (immigrant subject to deportation order, or non-immigrant) and characteristics of the conduct (driving while impaired by alcohol, or not). The phase 1 findings informed the design of the vehicular manslaughter vignettes used in the subsequent experiment^2^.

### Phase 1: Constructing the Experimental Vignettes

Using the Minneapolis Star Tribune, the largest newspaper in Minnesota^3^, we gathered 600 articles that met our criteria for potentially being about a homicide^4^. We screened the articles for relevance and established a 3-month cut point for analysis, leaving us with a final corpus of 177 test articles. We examined a 3-month period (March 18, 2019–June 18, 2019) in which we coded 110,250 words of text in 177 articles, covering 83 separate cases and 93 victims (seven cases involved multiple victims) of homicide.

We collected metadata about each article including date of publication, article title, author, and total word count. We also collected case-level information about the number of actors, the type of killing, any specific homicide-related charges, and the location of the incident. Finally, we also collected victim-level and offender-level information like age, gender, race, and the relationship between victim and offender.

We used the information gleaned from the corpus of 177 news articles to design our experimental vignettes. In our population of articles, victim and offender gender were mentioned a vast majority of the time (86.44 and 85.31% of the articles, respectively). The age of the offender was also usually mentioned (79.66% of articles), though the age of victims was reported only about half the time (53.11% of articles). It was much less common for race to be mentioned in the article with offender race mentioned around 17.51% of the time and victim race mentioned 18.64% of the time. Consequently, in our manufactured vignette we opted to report both victim and offender gender, offender age and one victim’s age, and no race information.

The most common type of killings reported in this period were shootings (42) and vehicular manslaughter (24). While we considered selecting shootings for our vignettes, we instead chose vehicular manslaughter because it lacks many confounding characteristics of other homicide types. In vehicular manslaughters there are less frequently pre-existing relationships between parties, neighborhood effects, or complicated motives that might not be clear from a news article in vehicular homicide cases. The fact that nearly 1/4 of homicides in the 3-month period were vehicular indicated that this time of crime would be plausible in the Minnesotan context. Importantly, vehicular manslaughter can also be framed as purely accidental or as accidental with compounding factors which gave us more flexibility in designing the vignettes.

In conducting a close code of all 177 articles we were also able to familiarize ourselves with the verbiage used in reporting about vehicular manslaughter. To replicate actual news stories as closely as possible, we selected two articles which formed the basis for our experimental vignettes (see Supplementary Material). We designed three vignettes derivative of the same vehicular manslaughter scenario (see Supplementary Material). The scenarios are as similar as possible in wording and keep offender and conduct characteristics constant excluding the key experimental manipulations. In the first scenario, we offered the basic information about the criminal event and use this as our control scenario. In the second scenario, we added information about the perpetrator having an elevated blood-alcohol content level and history of drunk driving. In the final scenario, we omitted the alcohol related information, but instead informed the reader that the perpetrator was an immigrant who had entered the country illegally 10 years prior and was set to be deported^5^. Our goal in choosing these three experimental vignettes was to examine the effects of conduct (drunk driving) and denigrated group membership (immigrant unlawfully present) on blameworthiness and punishment.

### Phase 2: Deployment on Amazon Mechanical Turk

We conducted our survey on Amazon Mechanical Turk, requiring the 191 participating Turkers to have above a 95% HIT rating and to be located in Minnesota^6^. We further confirmed their presence in the state of Minnesota by collecting the first three digits of each Turkers zip code at the end of the survey. While not a perfect proxy for residency, restricting the geography of participants makes it substantially more likely that participants would have been exposed to Minnesota crime media. We confirmed this by asking if participants had ever read news stories about crime in Minnesota, to which only 2.84% of respondents indicated that they never had (see Table 1). Participants were asked to read one of the three randomly assigned experimental vignettes and respond to questions about punishment, news consumption, and demographics.

**TABLE 1:** News engagement descriptives (%).

	Read news	Read MN crime news	Watch TV news
Never	0.57	2.84	14.2
Rarely	10.8	20.45	30.11
Sometimes	36.36	39.2	22.73
Often	38.07	27.27	23.86
Always	14.2	10.23	9.09
*N*	176	176	176

#### Independent Variables

The key manipulated variable was the potential blameworthiness of the vehicular homicide offender. We used three scenarios to re-design the news vignettes: control, driving under the influence (DUI), and immigration. In each scenario we altered only the blameworthiness information, holding all other facts about the incident constant. In the control vignette, we gave only basic information about the nature of the accident and the outcome. In the DUI condition, we included information about the elevated blood alcohol content (BAC) level of the offender. In the immigration vignette, we included information about the immigration history of the perpetrator, specifically that they immigrated to the United States illegally as a minor many years ago.

We measured a variety of demographic and related variables including gender, educational attainment, income, age, race, Hispanic ethnicity, and political views. Participants in our study were more likely to be male (56.02%) than female (43.43%). Nearly half had a bachelor’s degree (46.59%) and 85.14% of them described themselves as white. Around 60% of the participants made between $35,000 and $100,000 per year and were between the ages of 25 and 44 (full descriptives can be found in Supplementary Material). Importantly, we also asked participants to indicate their political views using a sliding scale from 0 to 100, with 0 being very conservative and 100 being very liberal. The sample skewed slightly liberal with a mean response of 59.3, though the standard deviation was large (29.73).

#### Key Dependent Variable

The key dependent variable in this analysis is the extent of punishment assigned to the hypothetical offender. Each participant was shown a slider and asked to assign a number of years of punishment between 0 and 10. While the numbers may be conceptually meaningful, we also want to focus on the behavior inherent to the response pattern. That is, a selection of “10” means something beyond just 10 years of punishment, it means the maximum punishment allowable. We use duration of punishment as a measurable proxy for the idea of blameworthiness, that is, the idea that some perpetrators deserve more punishment than others even if the outcome of the criminal act is the same. In this study, we keep the outcome of the scenario constant, only varying factors that might affect the level of culpability on the part of the perpetrator.

We are reasonably confident in our assertion that we can interrogate perceptions of morality using years of suggested punishment due to internal validity checks undertaken in the study design. In addition to the punishment question described above, we also asked participants to indicate their perception of the moral character of the driver on a seven-point Likert scale. These morality assessments were 54.71% correlated with suggested years of punishment, suggesting substantial conceptual overlap. In a simple linear regression model predicting years of punishment using the morality assessment we found a strong significant relationship (*P* < 0.00) and an *R*^2^ value of 0.30 again suggesting significant overlap between the two measures (see Supplementary Material for additional details and tabular representations).

We also included several other measures in the survey in order to collect additional information to contextualize the punishment responses. We asked participants about their news consumption, specifically how often they read news articles, watch the news on television, and read Minnesota crime news articles specifically. We also surveyed participants about a recent police shooting case in Minnesota that dominated news headlines, both to give context to participants’ understanding of the news and some of their opinions about fairness and justice^7^.

## Results

Punishment duration varied greatly by conduct and characteristic (see Table 2). In the control vignette, which included information only about the event and not the driver, respondents chose a punishment duration of 5.37 years on a scale of 0–10. This regression to the mid-point makes sense, given the limited information. However, when exposed to the DUI vignette the respondents assigned the driver a more punitive 9.19 years of prison on average. Interestingly, participants assigned 7.54 years of prison in the illegal immigration condition, reflecting a judgment in between the control condition and the DUI condition.

**TABLE 2:** Suggested punishment duration.

	*N*	Mean	Standard deviation
Control	55	5.37	3.47
DUI	62	9.19	1.52
Immigrant	75	7.54	3.06

We estimated separate linear regression models for each vignette type in order to understand how demographic factors and self-identified political views may impact punishment evaluations (Table 3). We found that none of the demographic factors predicted punishment duration in the control vignette, which is not particularly surprising given that the vignette contained very little information to potentially evoke differential responses. In the DUI vignette, respondent political views had some directional effects that approached significance, but none of the provided demographic variables significantly predicted punishment duration. This is consistent with literature suggesting the drunk driving is unanimously disparaged. Finally, in the immigration vignette, we found that only self-identified political views had a significant impact on punishment duration (*p* < 0.01). As self-identified political views became more conservative, suggested punishment duration increased.

**TABLE 3:** Regression predicting years of punishment by vignette type.

Variables	Control	DUI	Immigrant
**Political views**	−0.01 (0.02)	−0.02+ (0.01)	−0.04** (0.02)
**Income**			
Less than 10,000	−0.13 (5.26)	−1.18 (1.21)	−1.63 (2.89)
200,000 or more	−0.79 (7.58)	−0.28 (1.43)	3.58 (2.68)
**Education**			
High school/GED	−0.03 (2.36)	1.68 (0.72)	3.41 (1.82)
Some college	−0.91 (1.32)	0.17 (0.50)	1.60 (1.03)
**Gender**			
Male	−1.03 (1.22)	−0.43 (0.41)	−0.56 (0.94)
**Race**			
Black	−4.07 (5.38)	2.09 (1.95)	4.01 (2.42)
White	−5.06 (4.52)	0.51 (1.36)	1.05 (1.92)
**Ethnicity**			
Hispanic	5.24 (3.82)	0.62 (1.61)	−0.42 (2.56)
**Age**			
20–24	−2.17 (3.64)	1.29 (1.26)	−1.48 (3.84)
60–64	−1.27 (5.14)	0.40 (2.20)	1.23 (4.63)
**Constant**	12.02 (7.82)	9.54 (1.67)	8.40 (3.20)
**# of observations**	54	60	73

+p < 0.10, *p < 0.05, **p < 0.01, ***p < 0.001.

*Reported as regression coefficients with standard errors in parentheses. Insignificant values redacted for visual clarity, see Supplementary Material.*

**FIGURE 1:**
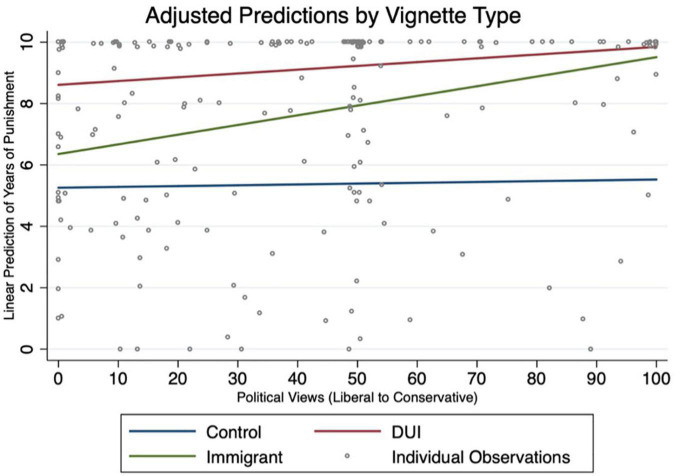
Adjusted predictions by vignette type.

In Figure 1, we plot the adjusted linear prediction of years of punishment by vignette type with a specific focus on political views, reversing the scale so that the left side of the *x* axis represents liberal identification and the right side represents conservative, for ease of visualization. We find that the slope of punishment across the control condition is flat across all ranges of political views. Consistent with our regression results, we see some effects of conservative political views on increased punishment in the DUI condition but find that suggested punishment in this condition is much higher all along the spectrum of self-identified political views. Also consistent with the regression results is the much larger positive slope in the immigration condition. In fact, at the furthest tail of self-identified conservative views predicted punishment duration scores in the immigration vignette and DUI vignette are not statistically different from each other. This means that the participants who self-identified as the most conservative perceived that an immigrant driver unlawfully present in the country who caused death deserved the same punishment enhancement as a drunk driver who caused death.

We report a tabular representation of these average marginal effects in Table 4, showing the ranges of confidence intervals for each vignette type.

**TABLE 4:** Average marginal effects by vignette type, political views.

	dy/dx	Standard error	*P* > | t|	95% confidence interval	
Vignette type					
Control	0.003	0.013	0.831	−0.022	0.027
DUI	0.012	0.013	0.323	−0.012	0.037
Immigrant	0.032	0.011	0.003	0.011	0.053

*N = 190.*

**FIGURE 2:**
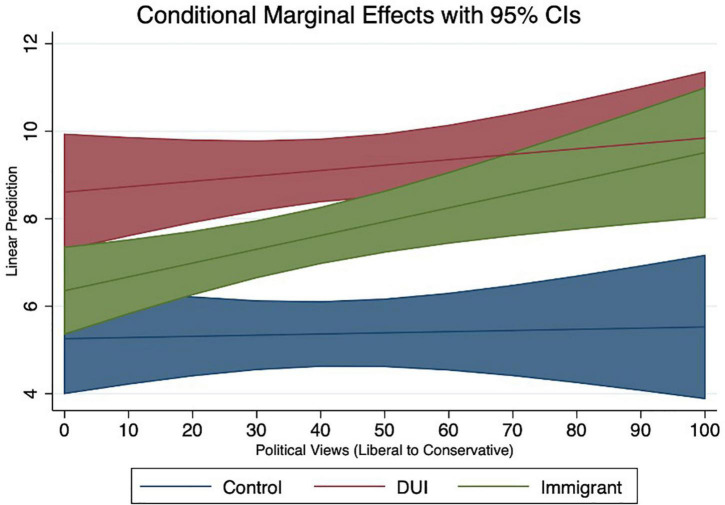
Conditional marginal effects of political views relative to control.

We also plot the conditional marginal effects of political views on linear predictions of punishment duration with a 95% confidence interval, confirming the results above (Figure 2). In this visual depiction behavior at the tails of the distribution is shown to be highly differentiated, with self-identified liberal views assigning punishment in the control and immigration conditions very similarly, while respondents with self-identified conservative views seemed to assign punishment more similarly between the DUI and immigration conditions.

## Discussion

Our results show that news consumers assign blameworthiness differently for the same criminal incident depending on what they learn about the conduct of the perpetrator (here, drunk driving) as well as the status of the perpetrator (here an immigrant present unlawfully). When we presented readers with the control vignette, which included no cues about immigration status or impaired driving, respondents selected punishment durations of a little over 5 years, reflecting moderately serious punishment. We argue that this relatively lower amount of punishment is reflective of a lack of moral cuing that was presented in the two other versions of the vignette. In the absence of any detail about circumstances, readers conceptualized the death as closer to an accident, because the perpetrator culpability is not specified by any moral characteristic of the person or the behavior. When we used predictive modeling, we found no significant demographic patterns in reader responses. This lack of influence of demographic characteristics suggests that we successfully retracted any moral cuing information from the control vignette that would prompt differential decision-making.

In contrast, in the DUI vignette, where we specify deviant behavior that has been entrenched as immoral (Schmidt, 2014) we see mean punishment substantially increased to more than 9 years of prison time. We want to stress that participants were not just choosing a particular number of years, rather they were selecting within a given range. That means that participants on average assigned close to the maximum amount of punishment allowed in this scenario. Once again, we do not find that any particular demographic characteristic is predictive of recommended punishment. This second set of null findings again conforms to findings in the literature indicating that drunk driving gives rise to moral outrage, and this response has become culturally pervasive enough to nullify potential group differences.

In the immigration vignette, we see something different, where there is substantial variation across participants regarding punishment and moral blameworthiness. As we demonstrate in Figure 1, readers with more liberal political views (closer to 0) selected a punishment duration much closer to the control condition, where readers with conservative views (closer to 100) selected a punishment duration much closer to the DUI condition. There are several components that we think might help explain this difference in punishment assignment. First, the issue of illegal immigration in the United States is in many ways a partisan issue with research postulating that this political entrenchment has grown in recent years (Dionne et al., 2008). Therefore, differential assignment of punishment by political views on a polarizing political issue is not altogether surprising. What is more interesting is the particular context in which it occurs. Importantly, there was nothing different about the conduct of the driver in the control vignette and immigrant vignette, yet the proscribed punishments were very different^8^. This implies that the same offense committed by someone without legal immigration status is perceived as more blameworthy than the same crime committed by someone who is not identified as lacking legal immigration status. This difference represents a very tangible consequence to differing interpretations of morality. This finding in particular merits future study to understand how political views may impact ultimate consequences for defendants in the criminal justice system, especially lawyers, judges, and laypeople involved in the justice system (i.e., juries) may bring their political ideologies into the courtroom.

Importantly, we did not assign an ethnicity to the driver, but rather only noted that he immigrated illegally as a minor many years ago. This likely presents a race cue of some kind, so the immigration could be proxying for racial resentment which has been shown to impact beliefs about illegal immigration (Hainmueller and Hopkins, 2014). Another possibility is that the difference in punishment is measuring the distinct but related concept of xenophobia.

These possibilities are especially salient in the Minnesotan context. The largest two immigrant communities in Minnesota are from Mexico (about 64,500 foreign-born Minnesotans) and Somalia (about 33,500 foreign-born Minnesotans) (Minnesota State Demographics Center [MSDC], 2018). So, the blameworthiness differences we observe might result from anti-Mexican racism and/or a version of anti-Black racism. In our study we collected that our Minnesota participants were conscious of race and national origin around the time they participated in this survey.

To get a sense of how participants understood crime and culpability in their community, after responding to the experiment vignette we asked them if they were familiar with the recent case of Mohamed Noor and Justine Damond. This case made headlines when Noor, an immigrant Somali police officer, mistakenly shot the unarmed Australian native Justine Damond who had called 911 to report a suspected sexual assault. Noor was found guilty of third-degree murder and manslaughter and sentenced to 12.5 years in prison, a marked difference in criminal justice outcomes compared to other police officers who killed civilians (Jackson, 2019). Notably, 1 year earlier, Minneapolis officer Jeronimo Yanez was acquitted of the Killing of Philando Castile (Jackson, 2019). When asked if they were familiar with the Noor case, 58.12% of participants said they were at least a little familiar. When asked about whether or not the verdict was fair participants were divided (34.74% believed it was fair, 12.11% believed it was not fair, and 53.16% were not sure) and themselves brought up the issues of race and immigration status. One respondent wrote:

“The facts in that case were not significantly different than other cop involved shootings in which the cop was exonerated. There was a feeling of racial undertones to the conviction.”

This represents a common theme among respondents: not necessarily a belief that Noor was innocent, but rather than inequality in the criminal justice based on race led to an unfair overall outcome. Participants struggled to choose a dichotomous marker of “fair” but were able to articulate agreement with a guilty verdict – without endorsing the broader system of punishment.

Another respondent compared the Damond case directly to the case of Castile saying:

“I think he should do SOME time, but not that much. Yes, he killed her. He didn’t listen to her. He didn’t follow training or protocol. However, other cops in the TCs (Twin Cities) have shot black, Hmong, Indian people, etc., and were not sentenced. If this cop is getting 12.5, the one that shot Philando Castile should have gotten 25.”

This respondent carefully articulates a disparity in blameworthiness relative to other cases that they conceptualize as similar. That is not to say that respondents were all in agreement. Many focused-on Noor as “trigger-happy” or articulated a belief that police officers should be held to a higher standard. Specific mentions of race or immigration status were generally avoided by participants who positively endorsed the outcome of the case, excluding one participant who suggested that:

“In my opinion he should have been deported back to his country with no chance of reentry.”

These responses demonstrate patterns in assessing blameworthiness mentally – but also in articulating blameworthiness around race. Further testing with a similar vignette design could more directly test these possibilities.

This study is limited in its generalizability given our focus on to vehicular homicides in the state of Minnesota. Future research should expand crime types and social contexts to examine whether these patterns are stable. Additionally, this analysis also only makes use of varying information about the offender (driver). Future work should consider varying the victim characteristics to more effectively measure the dyadic bias potentials between victim and offender.

This study advances knowledge about the role of news media in constructing popular perceptions moral guilt. All the scenarios we presented here were derivative of the same set of base facts. Moreover, both factors tested might have been present, simultaneously, about the actual incident, and the decision about whether and how to include either aspect in the story would be in the discretion of the writer. In other words, just because a driver had an elevated BAC level does not guarantee a news article reports on it, which may change the guilt perception of the perpetrator in that case. Evoking Schudson (2011), we do not mean to suggest that intentional misrepresentation by news writers causes distorted perceptions. Rather, a different portrayal of the truth for any number of reasons (unknown facts, facts perceived to be uninteresting or not newsworthy, limits on length, etc.) can change the contents of news unbeknownst to news readers. In the case of our sample, nearly all had read crime news before and a vast majority in the specific context of Minnesota. This ubiquity further explains the amplified importance of context in crime news. Even if news readers are not called to make direct decisions about a particular crime they read about in the news, the cumulative consequences of news can lead to racial stereotyping, fostering inaccurate fear of crime, and reifying mis-perceptions of who commits crime do affect everyone in society (Barlow et al., 1995; Gilliam et al., 1996; Sorenson et al., 1998; Thorson, 2001; Boulahanis and Heltsley, 2004).

## Conclusion

The construction of news stories can substantially influence readers’ judgments about blame and punishment for vehicular homicide offenders. By varying moral cues from neutral to negative in the same scenario, we demonstrate that readers select punishments around the mid-point when they lack information and select higher levels of punishment for universally condemnable moral behavior like drinking and driving. When faced with a morally controversial piece of information, like immigration status, we find that readers with differing political views assign different amounts of punishments. This finding underscores the importance of how news writing and presentation matters and how its influence can vary sharply according to pre-existing moral and political commitments of the reader.

## Data Availability Statement

The raw data supporting the conclusions of this article will be made available by the authors, without undue reservation.

## Ethics Statement

The studies involving human participants were reviewed and approved by the Northwestern University IRB. The patients/participants provided their written informed consent to participate in this study.

## Author Contributions

KA: conceptualization, design, data collection, data analysis, writing, and revising. JN: conceptualization, data analysis, writing, and revising. Both authors contributed to the article and approved the submitted version.

## Conflict of Interest

The authors declare that the research was conducted in the absence of any commercial or financial relationships that could be construed as a potential conflict of interest.

## Publisher’s Note

All claims expressed in this article are solely those of the authors and do not necessarily represent those of their affiliated organizations, or those of the publisher, the editors and the reviewers. Any product that may be evaluated in this article, or claim that may be made by its manufacturer, is not guaranteed or endorsed by the publisher.

## Footnotes

1 Studies often focus instead on cities that have the most homicide, ostensibly to get a robust picture of homicides overall (see Lattimore, 1997). In our case, we are less interested in homicide as a nationwide phenomenon, so we take this opportunity to focus on an understudied context.

2 Not all vignette-based work requires as much content analysis and adherence to real-world scenarios as we conducted here. However, in this case, the localized nature of the research required us to replicate reality as closely as possible to approximate news articles with appropriate verbiage, content, and tone. Notably, 66.7% of participants reported reading crime news from Minnesota (the context modeled in the vignettes) sometimes, often, or always, demonstrating the likely familiarity of the participant pool with a particular type of crime news.

3 The Star Tribune has a daily circulation of 288,315, a Sunday circulation of 581,063, and a digital subscription rate of 50,000.

4 Using the World Access News Database, we used one inclusive Boolean search function gather articles (kill* OR homicide* OR slay* OR murder*).

5 Note that this detail modeled on an actual case in Minnesota (see Supplementary Material). In this case, Jose O. Vasquez-Guillen was later deported, and a stream of mainstream and partisan media described Vasquez-Guillen in various ways that highlighted his immigration status including referring to him as “Salvadoran man,” “undocumented,” and an “illegal alien with deportation order” in news headlines. Interestingly, other headlines referred to him more generally as a “St. Paul resident.”

6 In order to ensure data quality, we included a short series of questions asking participants about their familiarity with a real-life case, then them to explain what happened in that case in words, and then asked them to evaluate the outcome as fair/unfair/not sure. Nine participants were removed from the final analysis because they provided incompatible or non-sensical responses.

7 We also asked two alternative questions that measured similar concepts to the main punishment measure. We asked participants if they thought the driver should get more or less punishment than average (5-point Likert scale) and about the moral character of the driver (7-point Likert scale). We do not focus on these measures here, but find that they follow the same general patterns reported in Table 2 (below).

8 It is feasible that participants were concluding that someone without legal immigration status would not have a driver’s license, making their criminal circumstances worse. However, we feel it is unlikely that this consideration explains the large amount of increased punishment assigned primarily by self-identified conservatives.
