# Supplementary material for: Assigning Punishment: Reader Responses to Crime News
Source: Front Psychol. 2022 Feb 16;13:784428. doi: 10.3389/fpsyg.2022.784428 (PMC8888689; doi:10.3389/fpsyg.2022.784428)
Supplement: Supplementary file 1 [file Data_Sheet_1.docx]

Supplementary Material

# Supplementary Data

**Inspiration Articles and Developed Vignettes**

Article 1:

2 drivers in Minn., N. Dakota cross centerline; 4 left dead

Two vehicles crossed the centerlines of roadways in Minnesota and North Dakota, causing crashes and killing four people.

Three people in a van were killed when a 16-year-old girl driving an SUV crossed the centerline late at night on a road west of Fargo and struck the van head-on. The teen driver and two other passengers in the cargo van were injured in the collision that occurred about 11:35 p.m. Friday on Cass County Road 10 about 40 miles west of Fargo, according to the North Dakota Highway Patrol.

Traffic was rerouted for about five hours as law enforcement collected evidence and cleared the debris from the crash involving a Lincoln Aviator and Ford Econoline van that blocked the road. The van’s driver, Matthew Wipf, 40, of Tower City, N.D., was killed along with two of his passengers: Kathy Wipf, 43, also of Tower City, and Dorothy Decker, 46, of Ipswich, S.D., according to the patrol. Both women were in the rear seat.

Two others in the van were injured: Henry Decker, 50, who was in the front passenger seat, and Heidi Hoffer, 35, who was in the rear seat. Both were also from Ipswich. The teen driving the SUV was identified as Sophia Weshnevski of Buffalo, N.D. She was taken by air ambulance to a Fargo hospital with serious injuries, the patrol said.

In the other crash, a driver with a history of drinking and driving was jailed, accused of a felony, after he crossed the centerline on an Otsego road and triggered a four-vehicle crash that left another motorist dead, authorities said Brandon D. Pedrys, 26, of Elk River, is suspected of criminal vehicular operation in connection with the death Friday of a driver who was struck head-on on County Road 39 near NE. Page Avenue, according to the Wright County Sheriff’s Office. Sharon Veiman, 62, of Anoka, died at the scene.

In less than eight years, Pedrys has been convicted twice for drunken driving, once for drinking and driving under age 21, twice for underage drinking and once for drug possession.

Article 2:

Suspect, 19, charged in fatal crash

A 19-year-old St. Paul man has been charged with felony drunken driving for striking another car last week and killing the other driver, according to charges.

Jose O. Vasquez-Guillen was charged Wednesday in Ramsey County District Court with Criminal vehicular homicide in connection with the crash on April 3 south of the St. Paul Downtown Airport. Mark J. O’Gara, 52, of St. Paul, died at the scene.

Two days after the crash, Vasquez-Guillen, who had previously been ordered deported, left the Ramsey County jail and was arrested by U.S. Immigration and Customs Enforcement (ICE). He remains in federal custody in the Sherburne County jail.

Vasquez-Guillen, a citizen of El Salvador, entered the country illegally at age 15 in January 2016, and a federal judge seven months later in Dallas ordered him deported, ICE spokesman Shawn Neudauer said Thursday. The deportation order came after Vasquez-Guillen failed to appear for his immigration hearing.

The charges say that Vasquez-Guillen’s blood-alcohol content about two hours after the crash was 0.149, well above the legal limit for anyone 21 and older to drive in Minnesota. O’Gara leaves behind a wife, 10 children and six grandchildren.

**Developed Vignettes (Control, DUI, Immigration)**

3 Struck and Killed in Crash

Three people in a van were killed when a 26-year-old man driving an SUV crossed the centerline late last night striking the van head-on. The driver sustained minor injuries in the collision that occurred at about 11:35 p.m. Friday night and was later taken into custody.

The van’s driver, a 50-year-old man, was pronounced dead at the scene. Two women, the driver’s wife and her sister, were taken to the hospital in critical condition where they passed away early this morning. They leave behind 2 children and 4 grandchildren.

3 Struck and killed in crash

Three people in a van were killed when a 26-year-old man driving an SUV crossed the centerline late last night striking the van head-on. The SUV driver, who had previously been jailed for drinking and driving, sustained minor injuries in the collision that occurred at about 11:35 p.m. Friday night and was later taken into custody. The charges say that the driver’s blood-alcohol content about two hours after the crash was 0.2, well above the legal limit in Minnesota.

The van’s driver, a 50-year-old man, was pronounced dead at the scene. Two women, the driver’s wife and her sister, were taken to the hospital in critical condition where they passed away early this morning. They leave behind 2 children and 4 grandchildren.

3 Struck and killed in crash

Three people in a van were killed when a 26-year-old man driving an SUV crossed the centerline late last night striking the van head-on. The SUV driver, who had recently been ordered to be deported, sustained minor injuries in the collision that occurred about 11:35 p.m. Friday night and was later taken into custody.

He was found to have entered the United States illegally at age 16 and was ordered to be deported after missing an immigration hearing. He was arrested by ICE agents shortly after leaving the Ramsey County Jail.

The van’s driver, a 50-year-old man, was pronounced dead at the scene. Two women, the driver’s wife and her sister, were taken to the hospital in critical condition where they passed away early this morning. They leave behind 2 children and 4 grandchildren.

# Supplementary Figures and Tables

## Supplementary Table 1

| S1: Participant Descriptives | | | | |
| --- | --- | --- | --- | --- |
|  |  | N |  | % |
| Age |  |  |  |  |
| 15 to 19 |  | 4 |  | 2.09 |
| 20 to 29 |  | 63 |  | 32.98 |
| 30 to 39 |  | 64 |  | 33.51 |
| 40 to 49 |  | 35 |  | 18.32 |
| 50 to 54 |  | 12 |  | 6.28 |
| 55 or older |  | 13 |  | 6.81 |
|  |  |  |  |  |
| Gender |  |  |  |  |
| Female |  | 83 |  | 43.46 |
| Male |  | 107 |  | 56.02 |
| Other |  | 1 |  | 0.52 |
|  |  |  |  |  |
| Race |  |  |  |  |
| Asian |  | 10 |  | 5.26 |
| Black or African American |  | 10 |  | 5.26 |
| White or Caucasian |  | 164 |  | 86.32 |
| Other |  | 6 |  | 3.14 |
|  |  |  |  |  |
| Hispanic Ethnicity |  |  |  |  |
| No |  | 184 |  | 97.35 |
| Yes |  | 5 |  | 2.65 |
|  |  |  |  |  |
| Education |  |  |  |  |
| Less than high school diploma |  | 3 |  | 1.57 |
| High school diploma/GED |  | 17 |  | 8.9 |
| Some college/vocational school |  | 62 |  | 32.46 |
| Bachelor's degree |  | 87 |  | 45.55 |
| Post-baccalaureate degree |  | 22 |  | 11.52 |
|  |  |  |  |  |
| Income |  |  |  |  |
| Less than 10,000 |  | 17 |  | 8.99 |
| 10,000 to 14,999 |  | 9 |  | 4.76 |
| 15,000 to 24,999 |  | 18 |  | 9.52 |
| 25,000 to 34,999 |  | 13 |  | 6.88 |
| 35,000 to 49,999 |  | 39 |  | 20.63 |
| 50,000 to 74,999 |  | 44 |  | 23.28 |
| 75,000 to 99,999 |  | 33 |  | 17.46 |
| 100,000 or more |  | 16 |  | 8.47 |
| N=189-191 due to non-response | | | | |

## Supplementary Table 2

| S2: Linear Regression Predicting Years of Punishment using Morality Assessment | | | | | | | | | | | |
| --- | --- | --- | --- | --- | --- | --- | --- | --- | --- | --- | --- |
|  |  |  |  |  |  |  |  |  |  |  |  |
|  |  | Coeff |  | Standard Error |  | P>\|t\| |  | 95% Confidence Interval | |  | R2 |
| Years of Punishment | | -0.24 |  | 0.026 |  | 0.00 |  | -0.29 | -0.18 |  | 0.3 |
| N=191 | | | | | | | | | | | |

We tested whether or not years of punishment was a reasonable measure of morality by comparing it directly to the results of a morality perception question included in the survey. Participants were asked how they perceived the drivers’ moral character via a seven-point Likert scale including: very bad, somewhat bad, slightly bad, neither good nor bad, slightly good, somewhat good, very good.

We then tested the relationship between the morality assessment and the years of punishment using pairwise correlation and simple linear regression. The concepts were significantly correlated at ~54%. The results of the simple regression model below indicate that there is a statistically significant relationship between the concepts (p<0.00) with a substantial r-squared value of 30%.

## Supplementary Table 3

**S3: Full Regressions**

| Appendix Table 2: Full Regression Models | | | | | | |
| --- | --- | --- | --- | --- | --- | --- |
|  |  |  |  |  |  |  |
| **Variables** |  | Control |  | DUI |  | Immigrant |
|  |  |  |  |  |  |  |
| **Political Views** |  | -0.01 |  | -0.02+ |  | -0.04** |
|  |  | (0.02) |  | (0.01) |  | (0.02) |
| **Income** |  |  |  |  |  |  |
| Less than 10,000 |  | -0.13 |  | -1.18 |  | -1.63 |
|  |  | (5.26) |  | (1.21) |  | (2.89) |
| 15,000 to 24,999 |  | 0.14 |  | -3.63 |  | -1.07 |
|  |  | (5.31) |  | (1.50) |  | (2.26) |
| 25,000 to 34,999 |  | 4.09 |  | -2.06 |  | -0.10 |
|  |  | (5.25) |  | (1.87) |  | (2.05) |
| 35,000 to 49,999 |  | 0.08 |  | -0.10 |  | 1.10 |
|  |  | (5.27) |  | (1.18) |  | (1.97) |
| 50,000 to 74,999 |  | 0.58 |  | 0.30 |  | -0.09 |
|  |  | (5.27) |  | (1.23) |  | (1.98) |
| 75,000 to 99,999 |  | 1.73 |  | -1.59 |  | 1.84 |
|  |  | (5.25) |  | (1.28) |  | (2.10) |
| 100,000 to 149,999 |  | -3.78 |  | 0.96 |  | 1.61 |
|  |  | (6.72) |  | (1.52) |  | (2.24) |
| 200,000 or more |  | -0.79 |  | -0.28 |  | 3.58 |
|  |  | (7.58) |  | (1.43) |  | (2.68) |
| **Education** |  |  |  |  |  |  |
| High school/GED |  | -0.03 |  | 1.68 |  | 3.41 |
|  |  | (2.36) |  | (0.72) |  | (1.82) |
| Less than HS |  | 0.50 |  | - |  | - |
|  |  | (3.56) |  | - |  | - |
| Post-baccalaureate |  | -1.09 |  | -1.26 |  | 0.87 |
|  |  | (2.67) |  | (0.59) |  | (1.42) |
| Some college |  | -0.91 |  | 0.17 |  | 1.60 |
|  |  | (1.32) |  | (0.50) |  | (1.03) |
| **Gender** |  |  |  |  |  |  |
| Male |  | -1.03 |  | -0.43 |  | -0.56 |
|  |  | (1.22) |  | (0.41) |  | (0.94) |
| Trans/gender non-conforming | | 1.76 |  | - |  | - |
|  |  | (4.93) |  | - |  | - |
| **Race** |  |  |  |  |  |  |
| Asian |  | -1.93 |  | - |  | - |
|  |  | (4.78) |  | - |  | - |
| Black |  | -4.07 |  | 2.09 |  | 4.01 |
|  |  | (5.38) |  | (1.95) |  | (2.42) |
| White |  | -5.06 |  | 0.51 |  | 1.05 |
|  |  | (4.52) |  | (1.36) |  | (1.92) |
| American Indian/Alaskan Native | | - |  | 4.46 |  | 4.89 |
|  |  | - |  | (1.94) |  | (3.77) |
| Other |  | - |  | -3.94 |  | 3.61 |
|  |  | - |  | (1.94) |  | (4.01) |
|  |  |  |  |  |  |  |
| **Hispanic** |  | 5.24 |  | 0.62 |  | -0.42 |
|  |  | (3.82) |  | (1.61) |  | (2.56) |
| **Age** |  |  |  |  |  |  |
| 20 to 24 |  | -2.17 |  | 1.29 |  | -1.48 |
|  |  | (3.64) |  | (1.26) |  | (3.84) |
| 25 to 29 |  | -3.36 |  | 0.90 |  | 0.46 |
|  |  | (3.82) |  | (1.16) |  | (3.86) |
| 30 to 34 |  | -0.18 |  | 0.06 |  | -2.20 |
|  |  | (3.61) |  | (1.20) |  | (3.95) |
| 35 to 39 |  | -1.03 |  | 0.93 |  | -1.38 |
|  |  | (3.63) |  | (1.21) |  | (4.04) |
| 40 to 44 |  | -2.96 |  | 0.95 |  | 0.08 |
|  |  | (3.82) |  | (1.26) |  | (4.08) |
| 45 to 49 |  | -6.04 |  | 0.50 |  | -1.84 |
|  |  | (5.20) |  | (1.22) |  | (4.03) |
| 50 to 54 |  | 2.26 |  | 0.05 |  | 0.61 |
|  |  | (4.29) |  | (1.44) |  | (3.98) |
| 55 to 59 |  | 1.41 |  | 0.94 |  | -0.22 |
|  |  | (5.37) |  | (1.74) |  | (4.17) |
| 60 to 64 |  | -1.27 |  | 0.40 |  | 1.23 |
|  |  | (5.14) |  | (2.20) |  | (4.63) |
| 70 to 74 |  | - |  | - |  | -0.89 |
|  |  | - |  | - |  | (4.38) |
|  |  |  |  |  |  |  |
| **Constant** |  | 12.02 |  | 9.54 |  | 8.40 |
|  |  | (7.82) |  | (1.67) |  | (3.20) |
|  |  |  |  |  |  |  |
| **# of Observations** |  | 54 |  | 60 |  | 73 |

+ p<0.10, * p<0.05, ** p<0.01, *** p<0.001
